# Moral foundations and decisions to donate bonus to charity: Data from paid online participants in the United States

**DOI:** 10.1016/j.dib.2019.104331

**Published:** 2019-07-30

**Authors:** Trevor O'Grady, Donald Vandegrift

**Affiliations:** Department of Economics, The College of New Jersey, USA

**Keywords:** Other-regarding behavior, Prosocial behavior, Moral foundations, Moral intuitions, Charitable giving, Public goods games, Altruism, Matching gifts

## Abstract

We present novel data linking other-regarding behavior outside of a laboratory with a participant's moral foundations, demographics, and opinions/awareness of social problems. These data were originally collected for Study 2 of O'Grady et al. (2019). Anonymous, paid participants were recruited through the online labor market Amazon Mechanical Turk (MTurk). Mturk workers located in the United States and meeting MTurk's “Masters Qualification” were offered $0.50 to complete a short survey. We used the moral foundations questionnaire (MFQ) developed by Graham et al. (2009) to classify participants based on their moral intuitions. After participants completed the MFQ and six diversion questions about their opinions and awareness of current social problems, we measured other-regarding behavior through an incentivized experiment. Respondents were awarded a $1 bonus and the option to donate any part of their bonus to a charity with the promise of a matching donation made by the researchers. Participants could only donate to one of three predefined charities and charity options were randomly assigned to respondents within three separate data collection waves. In addition, the dataset contains detailed information regarding situational details of the survey task including survey date, time of day, duration between worker request and recruitment, survey completion time, and performance on attention checks.

**Table:** Specifications Table

Subject	Social Sciences (General)
Specific subject area	Intersection of Behavioral Economics and Moral Psychology
Type of data	Table Tabular data Raw data stored as comma delimited text files (.csv) and excel spreadsheets (.xlsx) Processed data stored in Stata format (.dta)
How data were acquired	Respondents were recruited from Amazon Mechanical Turk (AMT) online labor market and paid to complete a survey.
Data format	Raw Analyzed
Parameters for data collection	Amazon Mechanical Turk workers were restricted to be U.S. residents and had to satisfy Mechanical Turk's “Masters” qualification.
Description of data collection	Amazon Mechanical Turk workers were paid $0.50 to complete the survey via Qualtrics and were given an additional $1 bonus if attention checks were successfully passed. Those who recieved a bonus were also asked how much of their bonus, if any, they would like to donate to their preferred charity from a randomly assigned list of three options.
Data source location	United States
Data accessibility	Repository name: Mendeley Data Data identification number: 99rkdj8ms7 Direct URL to data: https://data.mendeley.com/datasets/99rkdj8ms7
Related research article	Trevor O'Grady, Donald Vandegrift, Michael Wolek, Gregory Burr, On the Determinants of Other-Regarding Behavior: Field Tests of the Moral Foundations Questionnaire, *Journal of Research in Personality. 81, 2019, 224–237.*

**Table:** 

**Value of the data** • These data are useful for understanding how individual characteristics (such as moral foundations), opinions, and situational circumstances predict observed (rather than self-reported) other-regarding behavior, specifically one's willingness to donate money to a predefined set of charities. Furthermore, the randomized assignment of charity sets allows researchers to investigate how these relationships are affected by the specific charities presented to each respondent. • Researchers interested in the determinants of charitable giving as well as researchers performing meta-analyses related to other-regarding behavior in economic games, the Moral Foundations Questionnaire, and Amazon Mechanical Turk data may all benefit from these data. This dataset also allows researcher to directly replicate the original study associated with this dataset. • The dataset not only includes donation decisions, but also a respondent's charity preference. The determinants of charity preference were not explored in the original paper associated with this dataset. There are also many potential determinants of charitable giving in the dataset that have yet to be explored (e.g. engagement in environmental issues). • Because respondents were required to be MTurk Masters workers and were paid a relatively high wage ($0.50 for approximately 5–10 minutes of work) this dataset offers a useful point of comparison for similar studies that do not use MTurk workers or ones that use MTurk workers but do not take the same measures to ensure data quality. Data quality can be measured by performance on attention checks in the dataset (some of which are part of the standard MFQ) as well as the internal consistencies of the responses to the MFQ questions. Furthermore, detailed raw data from Amazon Mechanical Turk can allow researchers to investigate whether responses and characteristics of respondents differ by the characteristics of the work request to better understand the external validity of Mechanical Turk data. • The dataset is large enough to allow various partitions to investigate heterogeneous relationships in the data without losing substantial statistical power.

## 1 Data

Raw data were generated in three separate waves by MTurk and Qualtrics. These data were downloaded and processed using Stata to create the final dataset used by O'Grady, Vandegrift, Burr and Wolek (2019) [1]. This section briefly describes the raw data files (.csv), the Stata processing script (.do), and final dataset (.dta, .csv).

## 2 Raw data

### 2.1 Amazon Mechanical Turk worker recruitment data

Data collection from Amazon's Mechanical Turk (MTurk) was done in 47 “batches” or requests for workers, all with the same parameters. Raw data from each batch are stored in separate CSV files named **Batch_*XXXXXXX*_batch_results.csv**, where *XXXXXXX* refers to the batch number assigned by MTurk. Specific batch numbers are reported below by wave.

• Wave A – 3250472, 3250493, 3250494, 3250495, 3253329, 3253330, 3253332, 3253334, 3253336, 3253338, 3253340, 3253341, 3253343, 3253344, 3253346, 3253347
• Wave B – 3306322, 3306324, 3306325, 3306326, 3306327, 3306328, 3306330, 3306332, 3307082, 3307149, 3307176, 3307204, 3307232, 3307469
• Wave C – 3500097, 3500677, 3501544, 3503095, 3507526, 3507650, 3507805, 3507971, 3508607, 3508769, 3509683, 3510524, 3510723, 3511164

### 2.2 Qualtrics survey data

Data from the Qualtrics survey are stored in three CSV files, one for each survey wave. We removed columns in which respondents voluntarily provided their email address to receive a follow-up email about the study and then resaved these files as Excel spreadsheets.

• Wave A – Study2a_June 5, 2018_15.41.xlsx
• Wave B – Study2b_July 18, 2018_16.09.xlsx
• Wave C – Study2c_February 6, 2019_11.26.xlsx

### 2.3 Analyzed data

Raw data across all waves were merged and processed using Stata. Unfinished surveys were dropped from the final dataset but completed surveys with failed attention checks were not. Demographic data are quantified as dummy variables, with the exception of age which was already numeric. We use responses to the Moral Foundations Questionnaire (MFQ) to create scales for care, fairness, loyalty, authority, and purity following Graham et al. (2009) [2]. Consistent with the literature on Moral Foundations Theory, we sum care and fairness scales to create an individualizing foundations score and sum loyalty, authority, and purity scales to create a binding foundations score. All of the data processing commands are stored in the Stata “do-file” **dataprep.do** and the final dataset is saved in both Stata format (**allwaves.dta)** and comma-delimited format (**allwaves.csv)**. Variable descriptions are located in the file **Variable Codebook.txt**.

## 3 Experimental design, materials, and methods

### 3.1 Mechanical Turk recruitment

591 unique workers from Amazon's Mechanical Turk (AMT) were paid $0.50 to complete a survey. Participants were recruited in three waves from May 25-June 4, 2018 (Wave A), July 17-18 2018 (Wave B), and January 10–27 2019 (Wave C). Workers were restricted to locations in the United States and were required to be Mechanical Turk Masters, a qualification assigned by Mechanical Turk to identify high-performing and reliable workers based on their history. A summary of each wave is presented in Table 1.

**Table 1:** Summary of data collection waves and possible charity choice sets presented to respondents.

Data Collection Wave	Start-End Date	Total respondents	Total passing attention checks	Charity Conditions	Charity 1	Charity 2	Charity 3
A	May 25-June 4 2018	139	118	1	Natural Resource Defense Council	St. Jude Children's Research Hospital	Save the Children
				2	World Wildlife Fund	United Nations Children's Fund	American Red Cross
				3	The Nature Conservancy	Disabled American Veterans Charitable Trust	Doctors Without Borders
B	July 17–18 2018	100	86	4	The Nature Conservancy	St. Jude Children's Research Hospital	Doctors Without Borders
				5	The Nature Conservancy	Veterans of Foreign Wars Foundation	Doctors Without Borders
C	January 10–27 2019	352	319	6	The Nature Conservancy	St. Jude Children's Research Hospital	Doctors Without Borders
				7	The Nature Conservancy	Veterans of Foreign Wars Foundation	Doctors Without Borders
				8	The Nature Conservancy	Disabled American Veterans Charitable Trust	Doctors Without Borders
**Totals**		**591**	**523**				

Notes: Participants were randomly assigned to one of two or three conditions within each wave only if they passed the attention checks. Surveys were otherwise identical.

While participants were recruited from AMT, the survey itself was administered through the Qualtrics online platform. Before proceeding to the survey, participants were able to see the following project description and instructions on the AMT interface:

We are conducting an academic survey about opinions, current events, and behavior. Select the link below to complete the survey. At the end of the survey, you will receive a code to paste into the box below to receive credit for taking our survey.

**Make sure to leave this window open as you complete the survey.** When you are finished, you will return to this page to paste the code into the box.

Note: We are interested in gathering independent observations for our survey. Therefore, we will only allow you to complete ONE HIT. We will also use a qualification to exclude workers who have taken one of our similar surveys in the past. Thank you!

### 3.2 Demographics and moral foundations questionnaire

At the start of the Qualtrics survey participants were thanked for participating in the study and assured that their answers are strictly confidential. Participants were first asked to fill in demographic information including their gender, age, highest level of education, ethnicity, range of household income, religious identity if any, and political party affiliation. Participants were then asked to fill out the 32 question MFQ (six questions for each of the scales and two attention check questions). The responses to the demographic questions and MFQ are summarized across conditions in Table 2.

**Table 2:** Summary of Demographic Variables and MFQ Scales for all conditions.

Wave	A			B		C			Totals
Condition	1	2	3	4	5	6	7	8	

*Demographic Variables*									
Female	31%	48%	50%	40%	40%	51%	54%	52%	48%
White	75%	83%	83%	81%	86%	84%	75%	78%	80%
Age	38.1	37.7	41.4	39.4	36.1	39.1	40.6	40.1	39.4
Bachelor's Degree	61%	50%	60%	53%	44%	56%	50%	53%	53%
Income > $50,000	44%	40%	60%	42%	49%	62%	43%	44%	49%
Liberal	36%	48%	38%	44%	47%	47%	54%	48%	47%
Agnostic or Atheist	67%	48%	38%	56%	51%	49%	55%	42%	50%
*MFQ Scales*									
a. Care (1–30)	20.3	22.6	21.5	20.8	21.5	22.5	22.6	22.3	22.0
b. Fairness (1–30)	22.5	23.3	22.6	21.7	20.2	23.1	22.8	22.4	22.5
c. Loyalty (1–30)	12.3	13.5	12.0	12.2	12.8	12.7	11.6	13.2	12.5
d. Authority (1–30)	14.4	15.2	14.0	15.4	15.5	14.9	14.0	16.5	15.0
e. Purity (1–30)	11.2	13.3	10.3	13.9	12.2	12.0	10.3	13.8	12.1
Individualizing (a + b)	42.8	45.9	44.1	42.5	41.6	45.6	45.4	44.7	44.5
Binding (c + d + e)	37.9	42.0	36.3	41.5	40.5	39.6	35.9	43.4	39.7
Observations	36	40	42	43	43	108	102	109	523

Notes: The table reports averages and percentages of variables for each condition. Answers from respondents that failed an attention check (n = 68) are not summarized in the table because they were not assigned to a charity condition.

### 3.3 Opinions and awareness of social problems

Following completion of the MFQ, but before being prompted for charitable donations, respondents were asked six questions regarding three different social problems and their level of engagement in these issues. These questions were included in the survey to divert participant attention from the connection between the MFQ and their eventual donation decision at the end of the survey. Responses to these questions were not used in the original study but may be of use in related research. Responses were measured either on a 5-point Likert scale (1 Strongly Disagree, 2 Disagree, 3 Neutral, 4 Agree, 5 Strongly Agree) or Yes/No. The questions are listed below:

1. Climate change, disappearing species habitat, and environmental degradation have caused a decrease in U.S. living standards. (Likert scale response)
2. I have read or watched three or more news items (magazine articles, newspaper articles, blog posts, television news stories) on climate change, disappearing species habitat, or environmental degradation the past week. (Yes/No response)
3. Emergency public health crises and weaknesses in the U.S. healthcare system reduce U.S. living standards and subject portions of the U.S. population to unnecessary hardship. (Likert scale response)
4. I have read or watched three or more news items (magazine articles, newspaper articles, blog posts, television news stories) on U.S. public health or the U.S. health care system in the past week. (Yes/No response)
5. Poverty and easily curable diseases reduce the life expectancy of citizens in less developed countries. (Likert scale response)
6. I have read or watched three or more news items (magazine articles, newspaper articles, blog posts, television news stories) on the problems of poverty and disease in less developed countries in the past week. (Yes/No response)

The average responses to these questions are summarized across conditions in Table 3.

**Table 3:** Summary of responses to social problem questions by condition.

Wave		A		B			C	
Conditions	1	2	3	4	5	6	7	8

*Please read the following statement and indicate your agreement or disagreement (0 Strongly Disagree - 4 Strongly Agree)*								
Climate change, disappearing species habitat, and environmental degradation have caused a decrease in U.S. living standards.	2.5	2.7	2.3	2.5	2.6	2.7	2.7	2.4
Emergency public health crises and weaknesses in the U.S. healthcare system reduce U.S. living standards and subject portions of the U.S. population to unnecessary hardship.	3.0	3.0	3.0	2.8	3.0	3.1	3.2	3.0
Poverty and easily curable diseases reduce the life expectancy of citizens in less developed countries	3.5	3.4	3.6	3.4	3.6	3.6	3.5	3.5
*In the past week, I have read or watched three or more news items (magazine articles, newspaper articles, blog posts, television news stories) on:*								
Climate change, disappearing species habitat, or environmental degradation	31%	29%	26%	31%	47%	41%	45%	33%
U.S. public health or the U.S. health care system	24%	29%	36%	24%	36%	36%	40%	30%
Problems of poverty and disease in less developed countries in the past week	17%	17%	12%	13%	13%	14%	22%	11%
Observations	36	40	42	43	43	108	102	109

Notes: The table reports average level of agreement or percentage of “Yes” responses for each question by condition. Answers from respondents that failed an attention check (n = 68) are not summarized in the table because they were not assigned to a charity condition.

### 3.4 Attention checks

Before respondents were assigned to the charity conditions and offered a $1 bonus, we ended the survey task for participants with responses which were deemed inattentive. We judged attentiveness based on three questions, two of which come directly from the MFQ. The first MFQ question is “When you decide whether something is right or wrong, to what extent is whether or not someone was good at math relevant to your thinking?” We eliminated participants that responded somewhat relevant, very relevant, and extremely relevant to this question. The second MFQ question asks respondents to indicate their level of agreement or disagreement with the statement “It is better to do good than to do bad.” We eliminated participants that responded slightly agree, moderately agree, and strongly agree to this question. Lastly, we included a question in which the respondent is asked to read a passage in which we explicitly tell the respondent the correct answer to the follow-up question. The passage seen by respondents reads:

Forests are complex ecosystems in which trees are the dominant flora. Forests occur whenever the ambient temperature rises above 10 °C (50 °F) in the warmer months. Precipitation annually has to typically exceed 8 inches. Depending on the local climate, different types of forests grow. This question is a test of your attention. Please answer 100 trees. Colder climates at higher latitudes are often dominated by conifers such as pines, spruces, and larches. These forests are called taiga or boreal forests. Moderate-latitude climates generally give rise to deciduous forests, which are primarily composed of species such as oak, elm, birch, maple, beech, and aspen.

Following the passage respondents are shown a picture of a deciduous forest and are asked: “Roughly how many trees are in this photo of a deciduous forest?” And are given the options: 99 or fewer; 100; 200; 300; 400; and 401 or more. Respondents that did not answer 100 trees were eliminated from the last part of the survey. In total, 523 out of 591 participants passed all three attention checks.

### 3.5 Economic game: matched donations to charity

Within each wave, participants who both completed the survey and passed the attention checks were randomly assigned to one of the charity conditions via Qualtrics. In each condition, they were presented with a list of three charities with their corresponding logos and asked to choose a preferred charity among the options. After selecting their preferred charity, they were informed on the same page that they were receiving a $1 bonus payment (in addition to their wage) which they could keep or donate any portion of to their preferred charity and that the researchers would match their donations. The exact language of the prompt was as follows:

Thank you for taking our survey. In addition to the $0.50 payment you will receive for completing the survey, we have allocated additional funds in our budget per completed survey response. This amount, or some fraction of it, can be donated to the preferred charity that you indicated above or kept for yourself as a bonus payment paid through MTurk. Furthermore, any donation you make will be doubled by the experimenters. For example, if you donate $0.40 and keep $0.60, we will send $0.80 to your preferred charity.

On the slider below, indicate how much of the additional $1.00 you would like to donate to your preferred charity. The remaining money will be submitted to you as a bonus payment paid through MTurk. For example, to donate the entire $1.00 (thereby sending $2.00 to your preferred charity), leave the slider at 1. To keep the full amount, move the slider to 0. To donate $0.50 (thereby sending $1.00 to your preferred charity) and keep $0.50 for yourself, move the slider to 0.50.

**Fig. 1:**
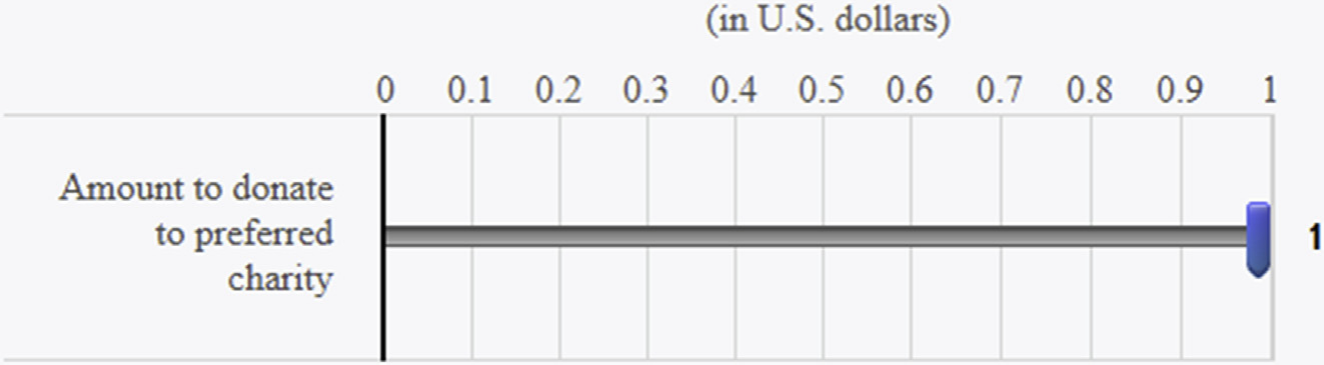
The figure shows the donation slider presented to respondents at the end of the Qualtrics survey.

The default position of the slider was set to $1 as pictured in Fig. 1 and could be moved in $0.01 increments.

To help assure participants that donations would be forwarded to their selected charities, we promised (on the same page) to set up a website showing the total amounts donated to each charity during the study and the receipts and thank you notes we subsequently received from the respective charities for each donation.^1^ We then offered participants the option to receive an e-mail (either through AMT or to an email address provided) with a link to the website. We further assured respondents that any email information they provided would not be shared with third parties or used for any other purpose. A summary of donations made by condition is reported in Table 4.

**Table 4:** Summary of donations by condition.

Wave	A			B		C			Totals
Condition	1	2	3	4	5	6	7	8	

*Donation Outcomes*									
Percent donating	64%	48%	50%	63%	44%	56%	56%	44%	52%
Donation amount	$0.26	$0.19	$0.20	$0.27	$0.17	$0.24	$0.26	$0.19	$0.22
Observations	36	40	42	43	43	108	102	109	523

Notes: The table reports averages and percentages of variables for each condition. Respondents had to pass an attention check in order to receive a bonus and be assigned to a charity condition.

## Footnotes

1 See: https://mfqdonationstation.weebly.com.
